# Insights into Ag-NPs-mediated pathophysiology and ultrastructural aberrations in ovarian tissues of darkling beetles

**DOI:** 10.1038/s41598-022-17712-z

**Published:** 2022-08-16

**Authors:** Lamia M. El-Samad, Mohamed A. Hassan, Nahed R. Bakr, Saeed El-Ashram, Eman H. Radwan, Karoline K. Abdul Aziz, Hussein K. Hussein, Abeer El Wakil

**Affiliations:** 1 Department of Zoology, Faculty of Science, Alexandria University, Alexandria, Egypt; 2 Protein Research Department, Genetic Engineering and Biotechnology Research Institute (GEBRI), City of Scientific Research and Technological Applications (SRTA-City), P.O. Box: 21934, New Borg El-Arab City, Alexandria Egypt; 3 Department of Zoology, Faculty of Science, Damanhour University, Damanhour, Egypt; 4 College of Life Science and Engineering, Foshan University, 18 Jiangwan Street, Foshan, 528231 Guangdong Province China; 5 Faculty of Science, Kafrelsheikh University, Kafr El-Sheikh, 33516 Egypt; 6 Department of Biological and Geological Sciences, Faculty of Education, Alexandria University, Alexandria, Egypt

**Keywords:** Biological techniques, Biotechnology, Environmental sciences

## Abstract

With the evolution of nanostructure materials, silver nanoparticles (Ag-NPs) emerged as the predominantly exploited nanomaterial in multifarious sectors due to their versatile properties. Along with the heightening applications of Ag-NPs, however, there is increasing concern over their indubitable toxicity towards the ecosystem, which indeed affects surrounding organisms and human health. In this study, we evaluated the detrimental effects of Ag-NPs in relation to Egyptian wild female beetles, *Blaps polychresta*, after injection with a single dose of Ag-NPs at different doses and monitoring for 30 days to determine the sublethal dose. Accordingly, the sublethal dose revealed the lowest negative influence was found at 0.03 mg/g body weight. The adverse impacts of Ag-NPs on the ovaries of female beetles were investigated by estimating the enzyme activities, DNA damage using a comet assay, and apoptosis by means of flow cytometry. Besides, the ultrastructural abnormalities were surveyed adopting transmission electron microscopy (TEM). The results manifested comet cells of 7.67 ± 0.88% and 22.33 ± 0.51 for Ag-NPs treated and control groups, respectively. Similarly, the data from flow cytometry demonstrated a substantial reduction in viable cells associated with a significant rise in apoptotic cells for the Ag-NPs treated group in comparison with the control group. Moreover, significant disturbances in enzyme activities for the treated group were perceived correlated with evident diminutions in antioxidant enzymes. Remarkably, the ultrastructural investigation emphasized these findings, exposing considerable deformities of the ovaries in the Ag-NPs treated group compared with the control group. To the best of our knowledge, this is the first report discussing the influence of Ag-NPs at the lowest dose on ovaries of *B. polychresta*. Collectively, our findings would significantly contribute to considering the critical effects of Ag-NPs at low levels, in addition to the potential use of *B. polychresta* as a good bio-indicator in ecotoxicological analyses.

## Introduction

Nanotechnology, which is fast advancing and generating materials with size-dependent key features, is projected to become the predominant origin for the transmission of nanomaterials (NMs). Given their particular physico-chemical inherent characteristics, such as greater durability, reactivity, or conductance, NMs might be employed to boost the advantages of a broad variety of products^1^. The substantial evolution of nanoparticles (NPs) gives rise to expansive exposure of humans to airborne NPs due to anthropogenic determinants, such as combustion engines and thermo-degradation of NPs^2^.

Among NMs, silver nanoparticles (Ag-NPs) have been acknowledged as forerunner materials in recent years owing to their intriguing traits, which seem to be distinctive from those of their larger counterparts^3^. The large surface area to volume percentage of Ag-NPs is considered the paramount property, which enables considerable implementation of the nanoparticle in multifarious sectors, including medical, electronic, and biotechnological applications^3,4^. Moreover, Ag-NPs were recently utilized as insecticide agents against white grubs (*Holotrichia* sp.), a potent pest of sugarcane in western Uttar Pradesh (India)^5^. These extensive applications reflect the accumulation and discharge of Ag-NPs into the ecosystem^6^. Intriguingly, one fifth of the total NMs established nowadays are silver-based, with an overall productivity of approximately 320 to 420 tons^7^. Exposure to Ag-NPs is inevitable owing to their pervasive utilization, which warrants safety assessments as well as a sounder awareness of any prospective effects on human safety^1^. Critically, the small size and high surface area of Ag-NPs facilitate their penetration through biological membranes, thereby augmenting their interaction with blood, and even fluid constituents of the lung membrane^8^. Moreover, the most detrimental mechanism stems from the drastic oxidation of Ag-NPs, discharging silver ions (Ag^+^), which incites changes in antioxidant enzyme defense, protein and DNA denaturation, lipid peroxidation, genotoxicity, and without doubt, the mortality of cells^2,9^.

Alarmingly, the exploitation of Ag-NPs might provoke unanticipated consequences in relation to their interaction with biological systems^8^. It has been reported that Ag-NPs could facilely infiltrate through the blood brain barrier (BBB) by transcytosis of capillary endothelial cells or move into other vital tissues^10^. The likely penetration of Ag-NPs to reproductive and developmental toxicity is crucial to consider since reproduction is an intricate process coordinated by several factors and it can be dislocated by subjecting to such hazardous nanoparticles, which menaces the survival of species, particular living organisms are more susceptible during developmental stages^11^.

Several studies demonstrated the agglomeration of Ag-NPs in the ovaries of rats after administering the females with diverse sizes of the nanoparticles, and the toxicity could migrate to the placenta, transferring to the offspring whether the administration was carried out orally or injected^12,13^. Moreover, the Ag^+^ was detected in different organs in the offspring, including the liver, kidney, and even the hippocampus^1,14^. Additionally, histopathological investigations of female rats administered Ag-NPs exhibited critical alterations, including apoptosis, and atretic and disintegrated follicles in the ovaries^15^.

In light of recent events in Ag-NPs, it is becoming extremely difficult to ignore their broad distribution and the consequences of their toxicity. Although prior studies have been devoted to investigating the prospective threats of Ag-NPs towards the environment and human beings, there is a relative paucity of empirical research, investigating the impact of Ag-NPs on the structure and physiological functions of ovaries in terrestrial organisms, particularly insects. In our recent studies, we highlighted a strong and consistent association between exposure to Ag-NPs and the structural and physiological alterations, even at the molecular and transcriptional level of insect gonads in darkling beetles (*Blaps polychresta*). However, it is well known that females are more susceptible to the toxicity of nanoparticles due to great variations in physiological functions^1,16,17^. A few recently published studies delineated the importance and potential exploitation of female beetles as nanotoxicity indicator species^3^. Herein, we ascertained the sublethal dose of Ag-NPs in wild female insects, *B. polychresta*, after injection with a single dose at various concentrations. The impacts of Ag-NPs on the ovaries in female beetles were then investigated in the group subjected to the sublethal dose after 30 days in relation to enzymatic activities, apoptotic and genotoxic influences. Moreover, to delve into other significant changes, the ultrastructural malformations of the ovaries were examined.

## Results

### Survival and mortality of *B. polychresta* after injection with Ag-NPs

**Figure 1:**
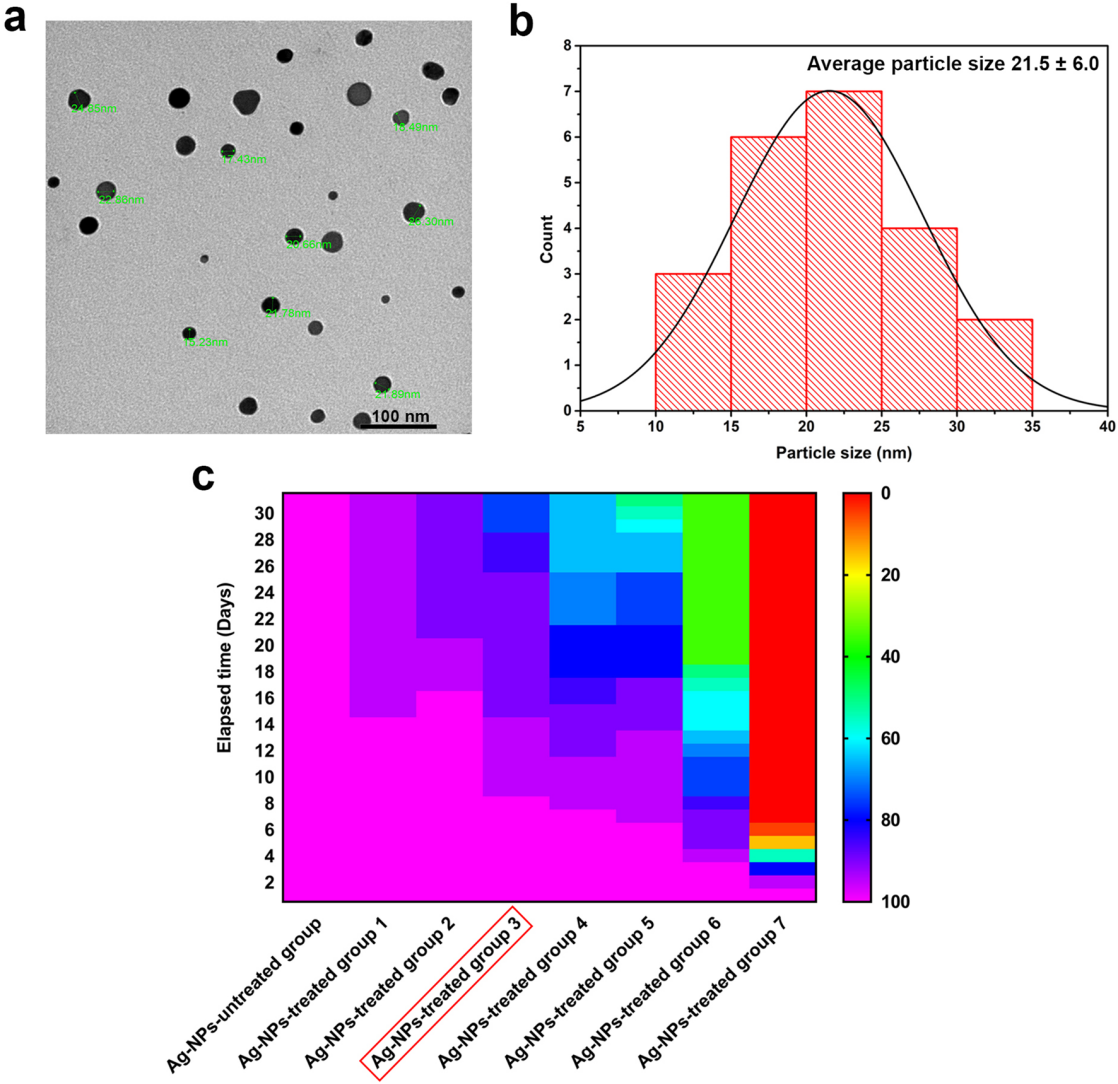
Characterization of Ag-NPs, (**a**) A transmission electron micrograph of Ag-NPs exhibits the size of the nanoparticles used in this study, and (**b**) The pore size distribution of Ag-NPs shows that the majority of nanoparticles are within a range from 20 to 25 nm. Fig. (**c**) The heat map presents the survival probabilities of different groups from 1 to 7 of female beetles, *B. polychresta,* throughout 30 days after injection with Ag-NPs at various doses of 0.01, 0.02, 0.03, 0.04, 0.05, 0.06, and 0.07 mg/g body weight for each group, respectively. The red rectangle signifies the group of beetles treated with Ag-NPs, which was selected for further studies in comparison with the control group.

Figure 1a,b portray that the diameters of Ag-NPs varied within a range from 5 to 26.30 nm with an average size of 21.5 ± 6.0 nm, and the particle size distribution exhibits that the most particle size emerged from 20 to 25 nm, which is in agreement with the certification furnished by the manufacturer, specifying that the size of Ag-NPs is less than 50 nm.

The survival rates of *B. polychresta* for the seven groups were determined after administration of a single Ag-NPs dose alongside the control group, implying the anticipated survival period of the beetles from Day 0 until day 30 as depicted in Fig. 1c. These findings clearly exhibit that Ag-NPs have a detrimental impact on beetle survival, with a full mortality effect for group 7 even during the first 5 days of the experiment. The results revealed the variations of Ag-NPs impact on the beetles on the basis of the concentration of the injected nanoparticles. Furthermore, Kaplan–Meier survival analysis substantiated these findings as shown in Fig. S1. According to the data obtained from the log-rank test for the entire groups, the highly significant influence of Ag-NPs could be perceived. Precisely, group 7 injected with the highest concentration of Ag-NPs (0.07 mg/g body weight) demonstrated a mortality ratio of 100%, whereas, group 5 treated with a dose of 0.05 mg/g body weight showed accumulative mortality of 50% at day 30, which implies that the latter dose is the LD50 for *B. polychresta* in this investigation. The comparison between group 1 and the other groups based on the survival rate following the log-rank test pointed out that the dose of 0.03 mg/g body weight could be considered as a sublethal dose. We thus investigated biochemical and histological characteristics of the ovarian tissues collected from the female beetles of group 3 (Ag-NPs-treated group 3) in comparison with the control group.

### Detection of Ag^+^ in ovarian tissues of *B. polychresta*

**Figure 2:**
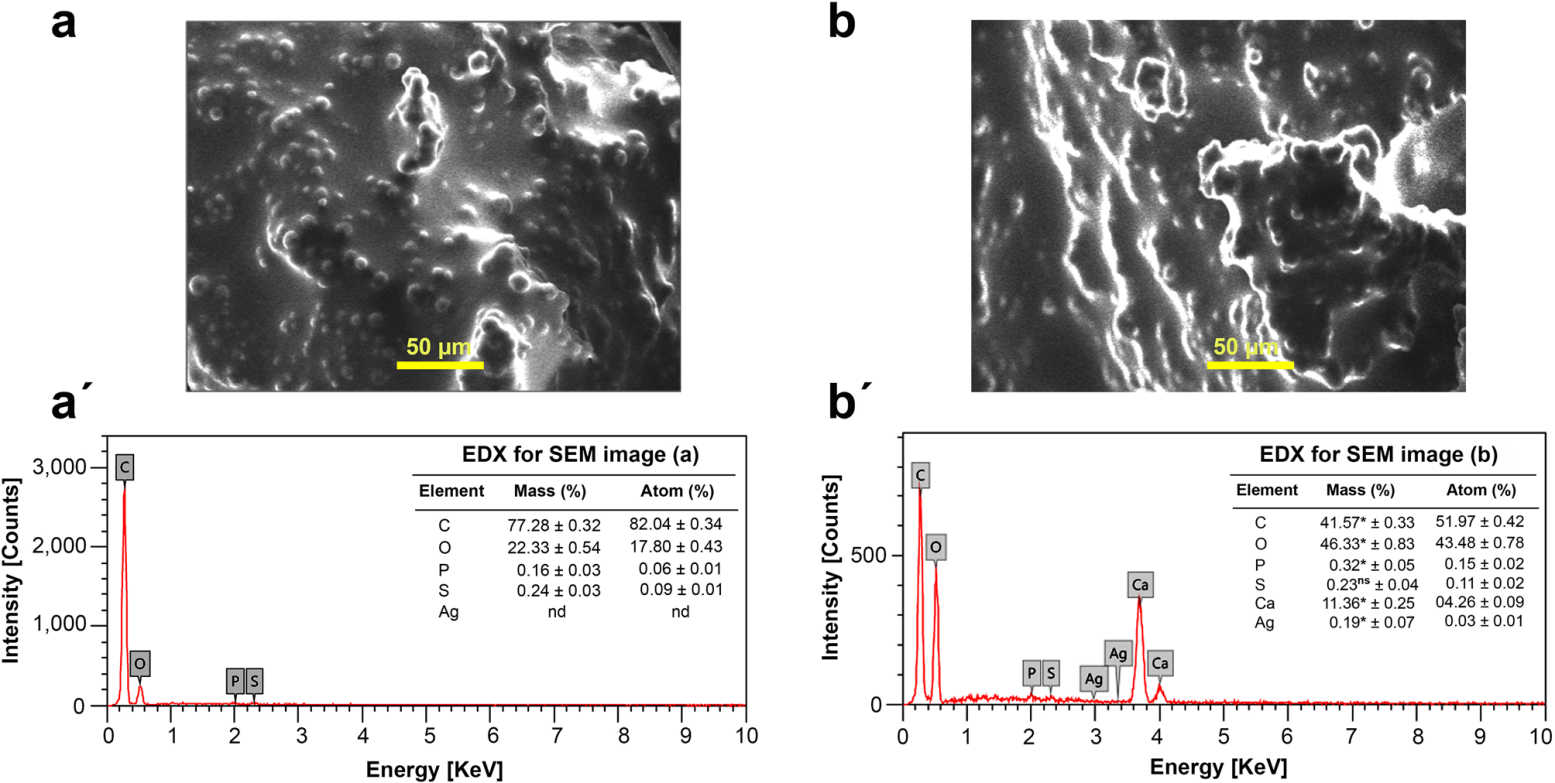
SEM of (**a**) ovarian tissues from the control group and (**b**) ovarian tissues from Ag-NPs-treated group 3. (**a'**) and (**b'**) EDX spectra of ovarian tissues harvested from the control and Ag-NPs-treated group, respectively, show quantitative analyses of the incident elements, indicating the presence of Ag^+^ in the Ag-NPs-treated group and calcium (Ca) in comparison to the control beetles. This could be derived from calcium dysregulation caused by increased levels of reactive oxygen species (ROS). The analysis was performed using three sections of ovarian tissues dissected from three beetles and the results are shown as mean ± SEM (*P ≤ 0.05, and (ns) indicates a non-significant difference).

It could be perceived from the SEM and energy dispersive X-ray micro-analyzer (EDX) analyses in Fig. 2a,a´, the incidence of four elements, including carbon (C), phosphorus (P), sulfur (S), and oxygen (O) in the investigated ovaries of insects in the control group. In the same manner, as shown in Fig. 2b,b´, the ovarian tissues of group 3 treated with Ag-NPs exposed the comparable elements in higher concentrations along with calcium (Ca), which might be attributed to the disorder effect of silver on the physiological functions of beetles. Moreover, the results revealed no distinctive peak of Ag^+^ was found in the case of the control group. On the contrary, in beetles of group 3 administered with a single dose of Ag-NPs (0.03 mg/g body weight), Ag^+^ was perceived in the ovaries after 30 days with the average ratio of 0.37 ± 0.07 after measuring the Ag^+^ content in the ovaries of 9 beetles.

To substantiate that the Ag-NPs are not only agglomerated in the ovarian tissues but also in other organs, we investigated the midgut tissues of group 3 treated with Ag-NPs. As depicted in Fig. S2, different elements with various concentrations were determined in control and the beetles were treated with Ag-NPs. Specifically, the presence of Ag^+^ peak in the beetles treated with Ag-NPs in contrast to the control group. Besides, it could be extrapolated that the critical disorder of the elements is a consequence of nanoparticle impacts.

### Biochemical assays in *B. polychresta* after treatment with Ag-NPs

**Figure 3:**
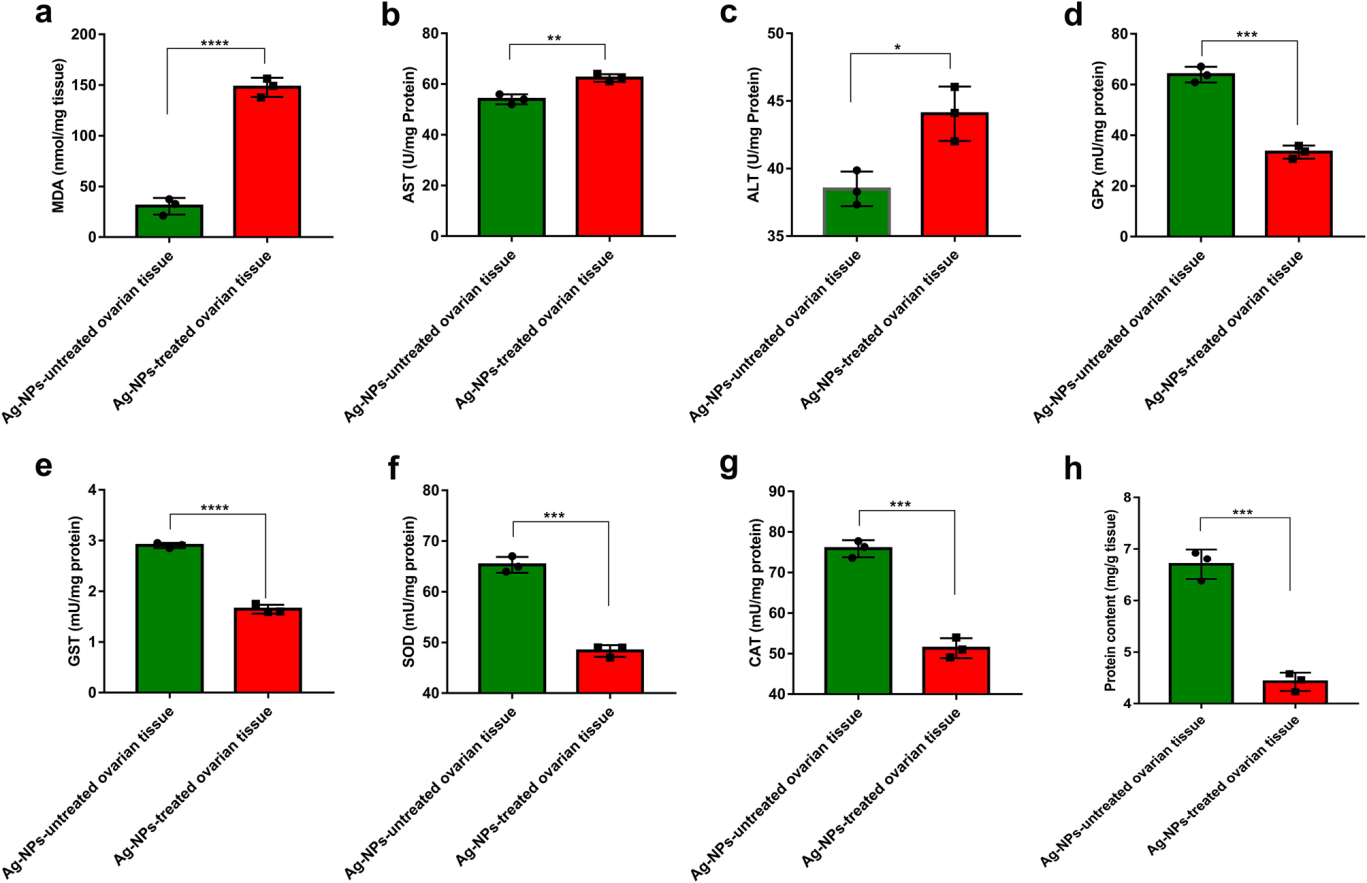
Biochemical assays of (**a**) Malondialdehyde (MDA), (**b**) Aspartate aminotransferase (AST), (**c**) Alanine aminotransferase (ALT), (**d**) Glutathione peroxidase (GPx), (**e**) Glutathione S-transferase (GST), (**f**) Superoxide dismutase (SOD), (**g**) Catalase (CAT), and (**h**) Protein content in the ovaries homogenates of Ag-NPs-treated group and the control group. The experiments were performed in six replicates and the results are shown as mean ± SEM (****P ≤ 0.0001, ***P ≤ 0.001, **P ≤ 0.01, *P ≤ 0.05). The results evince significant differences between the different parameters in the Ag-NPs-treated group in comparison with the control insects.

The biochemical parameters of the ovarian tissues in beetles exposed to a single dose of Ag-NPs (0.03 mg/g body weight) demonstrated significant dysfunctions after 30 days of treatment compared to the untreated beetles in the control group, as illustrated in Fig. 3. Figure 3a displays that the MDA content in the insect ovarian homogenate of group 3 was remarkably amplified in comparison with the MDA level of untreated insects. Likewise, the activities of AST and ALT in ovarian homogenate were significantly increased as a consequence of treatment with Ag^+^ as presented in Fig. 3b,c. In terms of the protein content, it was considerably lessened in the ovaries of group 3 compared with the control group (Fig. 3h). Figure 3d–g depict that the activities of the antioxidant enzymes functioning to control the overproduction of reactive oxygen species (ROS), including GPx, GST, SOD, and CAT were markedly lower in the ovarian tissues of group 3 after 30 days of treatment with Ag-NPs than in the control beetles. Overall, the treatment of *B. polychresta* with Ag-NPs engendered aberrant performances of biochemical functions in the beetles.

### Genotoxicity in *B. polychresta* after treatment with Ag-NPs using comet assay

**Figure 4:**
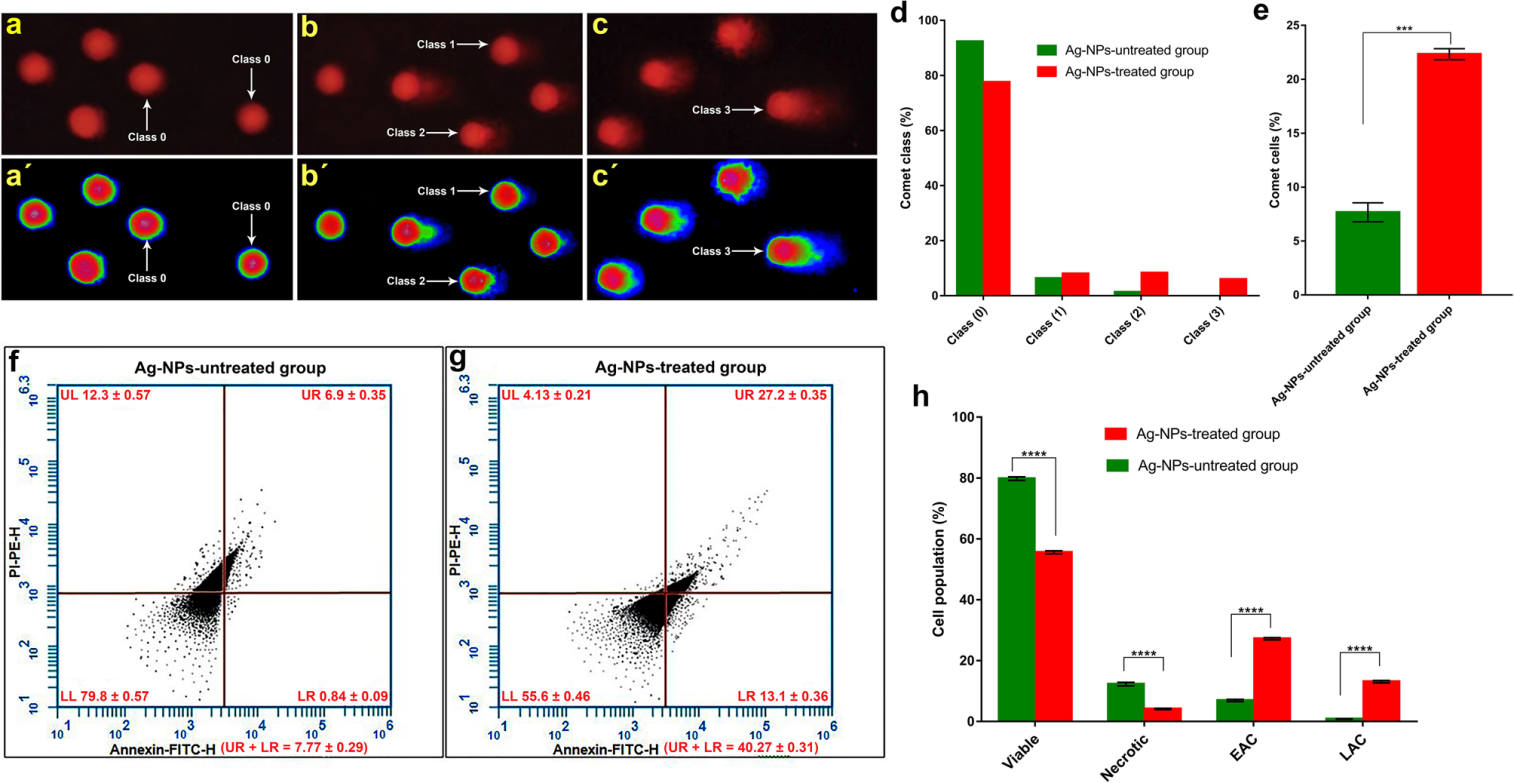
Evaluation of DNA damage following comet analysis in the ovarian tissues of *B. polychresta*, (**a**) a slide for cells from untreated beetles displays normal DNA (class 0), and (**b**–**c**) slides for cells from Ag-NPs-treated beetles show damaged DNA (class 1, class (2), and class 3), while (**a'**–**c'**) reveal the slides after analysis using Imagej Fiji software (https://imagej.net/software/fiji/downloads), demonstrating the tails of DNA. Fig. (**d**, **e**) illustrate the analysis of 300 cells taken for the comet assay, showing the different classes in (d) and the comet cells in (**e**). (**f**, **g**) exhibit the flow cytometry analyses of annexin-V-FITC and propidium iodide staining of the Ag-NPs-untreated group and the Ag-NPs-treated group 3, respectively, and (**h**) demonstrates the analysis of the flow cytometry results, showing the significant lessening in viable cells and a significant rise in apoptotic cells for the Ag-NPs-treated group 3 compared to the control group. The (UL) represents (PI + /Annexin V −), indicating necrotic cells, the (LL) represents (PI − /Annexin V −), which refers to normal cells, the (UR) is (PI + /Annexin V +), pointing to early apoptotic cells (EAC), and the (LR) alludes to (PI − /Annexin V +), showing late apoptotic cells (LAC). The data was analyzed using CellQuest Pro software version 5.2.1, 2005 (BD Biosciences, San Jose, CA). For comet analysis, five ovarian tissues from different beetles of Ag-NPs-treated group 3 and the control group were used for developing five slides and 100 comets per slide were investigated. Flow cytometric analyses were also performed using five ovarian tissues from different beetles of Ag-NPs-treated group 3 and untreated beetles. The results are presented as mean ± SEM (****P ≤ 0.0001, and ***P ≤ 0.001), indicating the significant differences between the Ag-NPs-treated group 3 and the control beetles in relation to genotoxicity and cellular toxicity.

The comet analysis exhibited critical damage to ovarian cell nuclei in *B. polychresta* injected with the Ag-NPs (group 3) in comparison with the untreated insects as displayed in Fig. 4a–e. According to the length of the DNA tails, the damage of DNA was classified into four classes from 0 to 3, in which class 0 refers to normal and intact DNA without any impairment, whereas class 3 alludes to the extensive damage of DNA in the nuclei of examined cells as shown in Fig. 4a–c and Fig. 4a'–c'. Furthermore, classes 1 and 2 point to the different interference degrees of DNA in the cells obtained from the ovaries of beetles. The outcomes expose that the ratios of nuclei without evident DNA damage (Class 0) were 92.3% for the control group and 77.6% for the group 3 (Fig. 4d). Consequently, the parentages of total comet cells were 7.67 ± 0.88% and 22.33 ± 0.51% for the control group and Ag-NPs-treated group 3, respectively, as shown in Fig. 4e. Specifically, for the control group, we found out the rest of the cells belonged to class 1 and class 2 with rates of 6.33% and 1.33%, respectively. Moreover, the upshots disclosed no nuclei with damage to be defined as class 3 in the control group. By contrast, in the group 3 treated with Ag-NPs, the percentages of nuclei with extensive devastation of DNA that could be classified as the third class was 6%, while 8% and 8.33% belonged to classes 1 and 2, respectively. Collectively, the comet assay evidently demonstrated the detrimental influence of Ag-NPs on *B. polychresta* in relation to genotoxicity*.*

### Cell viability assay by flow cytometry

In order to evaluate the viable, apoptotic and necrotic cells isolated from the ovarian tissues of Ag-NPs-treated group 3, the annexin V-FITC assay was carried out by means of flow cytometry as depicted in Fig. 4f–h. It is discernible from the data in Fig. 4h the significant difference of viable cells between the control group and Ag-NPs-treated group 3, where the viable cells were 79.8% for the control group, while the results exhibited viability cells of 55.6% for the treated insects with Ag-NPs. Furthermore, the results revealed statistical significant differences between the two examined groups with regards to the ratio of necrotic and apoptotic cells. Remarkably, the necrotic cells were significantly lessened in the group 3, recording 4.13 ± 0.21%, while the estimated percentage of necrotic cells in the control group was 12.30 ± 0.57%. Considering the apoptotic cells, they were significantly augmented in group 3 in both cases of early and late apoptotic cells as a result of treatment with Ag-NPs, reporting 27.2 ± 0.35% and 13.1 ± 0.36%, respectively. In contrast to these findings, the parentage of early apoptotic cells was 6.9 ± 0.35% in the untreated insects. Notably, the late apoptotic cells were only perceived to be 0.84 ± 0.09% in the control group. Taken together, the results of the annexin V-FITC assay emphasized the comet assay findings, highlighting the deleterious impact of Ag^+^ on *B. polychresta*.

### Ultrastructural alterations of ovaries from *B. polychresta* after treatment with Ag-NPs

**Figure 5:**
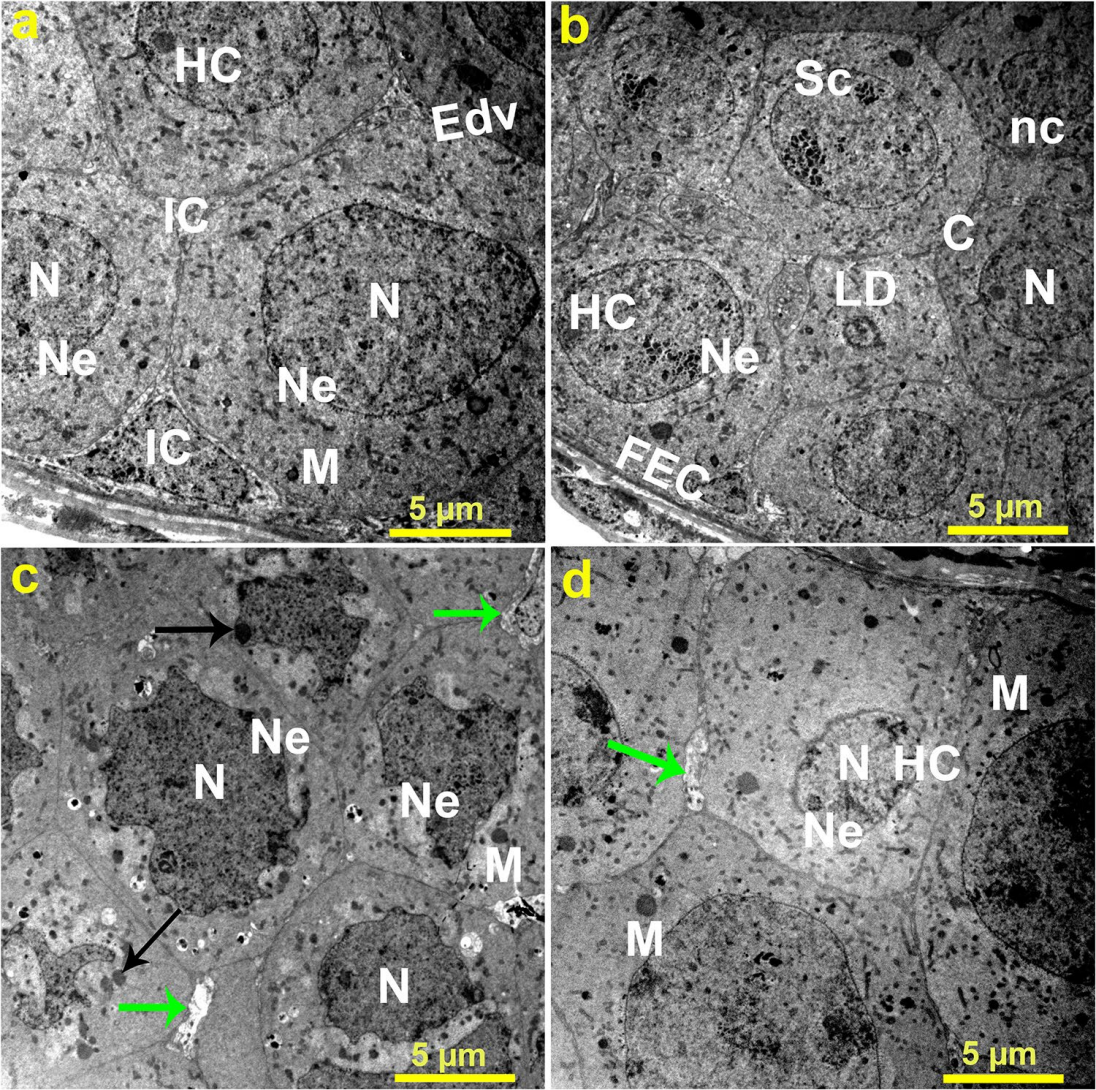
Transmission electron micrographs of (**a**, **b**) two views of trophocytes in the ovarian tissues of the control group exhibit normal trophocyte structures with rounded nuclei, cytoplasm, regular nuclear envelope, nurse cells, follicular layer with follicular epithelial cells, lipid droplets, patches of heterochromatin, synaptonemal complexes, and mitochondria, whereas (**c**, **d**) two views of trophocytes in the ovarian tissues of the Ag-NPs-treated group 3 show substantial abnormalities as a result of oxidative stress stemmed from the Ag-NPs, involving pyknotic nuclei, disintegrated nuclei, irregular nuclear envelope, malformed mitochondria, abnormal condensation of chromatin, and degenerated interstitial cells. Nucleus (N), nuclear envelope (Ne), heterochromatic (HC), mitochondria (M), lipid droplet (LD), interstitial cells (IC), cytoplasm (C), nurse cells (nc), follicular layer with follicular epithelial cells (FEC), synaptonemal complexes (Sc), electron dense vesicle (Edv), black arrows point to pyknotic nuclei, and green arrows indicate degenerated interstitial cells. Three ovarian tissues from different beetles of Ag-NPs-treated group 3 and the control group were inspected.

**Figure 6:**
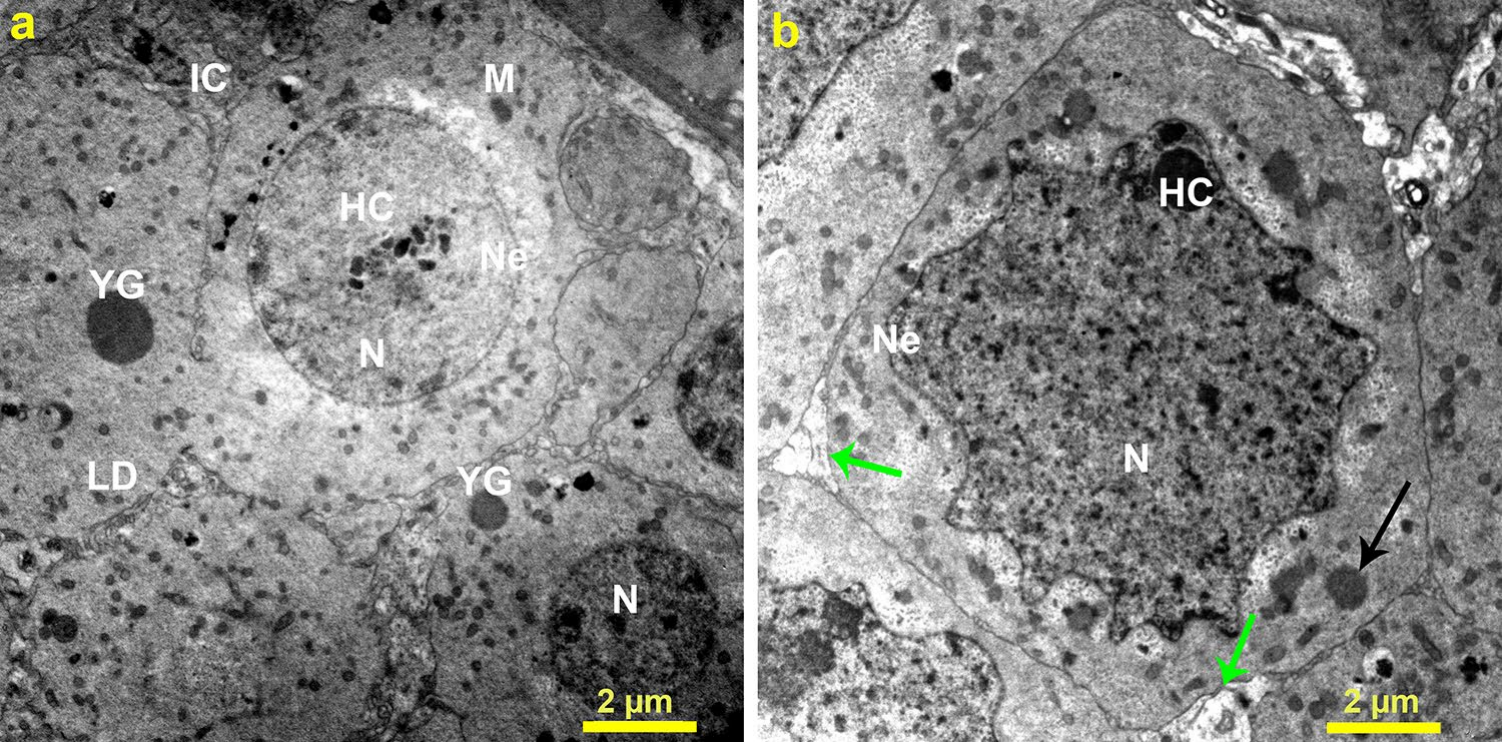
Transmission electron micrographs of (**a**) ovarian tissue of *B. polychresta* from the control group depicts typical structures, including spherical nuclei, regular nuclear envelope, heterochromatin, mitochondria, lipid droplet, yolk granules, and interstitial cells, and (**b**) ovarian tissue from the Ag-NPs-treated group 3 delineates different deformities, including degenerated nucleus, irregular nuclear envelope, pyknotic nuclei, and fragmented interstitial cells. Nuclei (N), nuclear envelope (Ne), heterochromatin (HC), mitochondria (M), lipid droplet (LD), yolk granules (YG), interstitial cells (IC), black arrows point to pyknotic nuclei, and green arrows indicate degenerated interstitial cells. Three ovarian tissues from different beetles of Ag-NPs-treated group 3 and the control group were used for TEM analysis.

To study the effect of Ag-NPs on the structure of ovaries harvested from *B. polychresta*, the ultrastructural alternations of ovaries from beetles (Ag-NPs-treated group 3) were inspected by means of TEM in comparison with the control group. TEM analysis of trophocytes in the control group emerged in normal structure as spherical cells, including typical nuclear envelopes associated with recognizable aggregation of chromatin in the nuclei as illustrated in Fig. 5a,b. Moreover, it could be perceived that the interstitial cells between the trophocytes have a regular structure, in addition to the entire systematic cytoplasmic organelles. In contrast to this structure, it could be detectable in the case of Ag-NPs-treated group 3 some deformed structures, including Pyknotic nuclei correlated with projected disintegration of mitochondria and interstitial cells (Fig. 5c, d). Correspondingly, the similar observations were found in the ultrastructure of ovaries from Ag-NPs-treated group 3 and Ag-NPs-control group as portrayed in Fig. 6.

**Figure 7:**
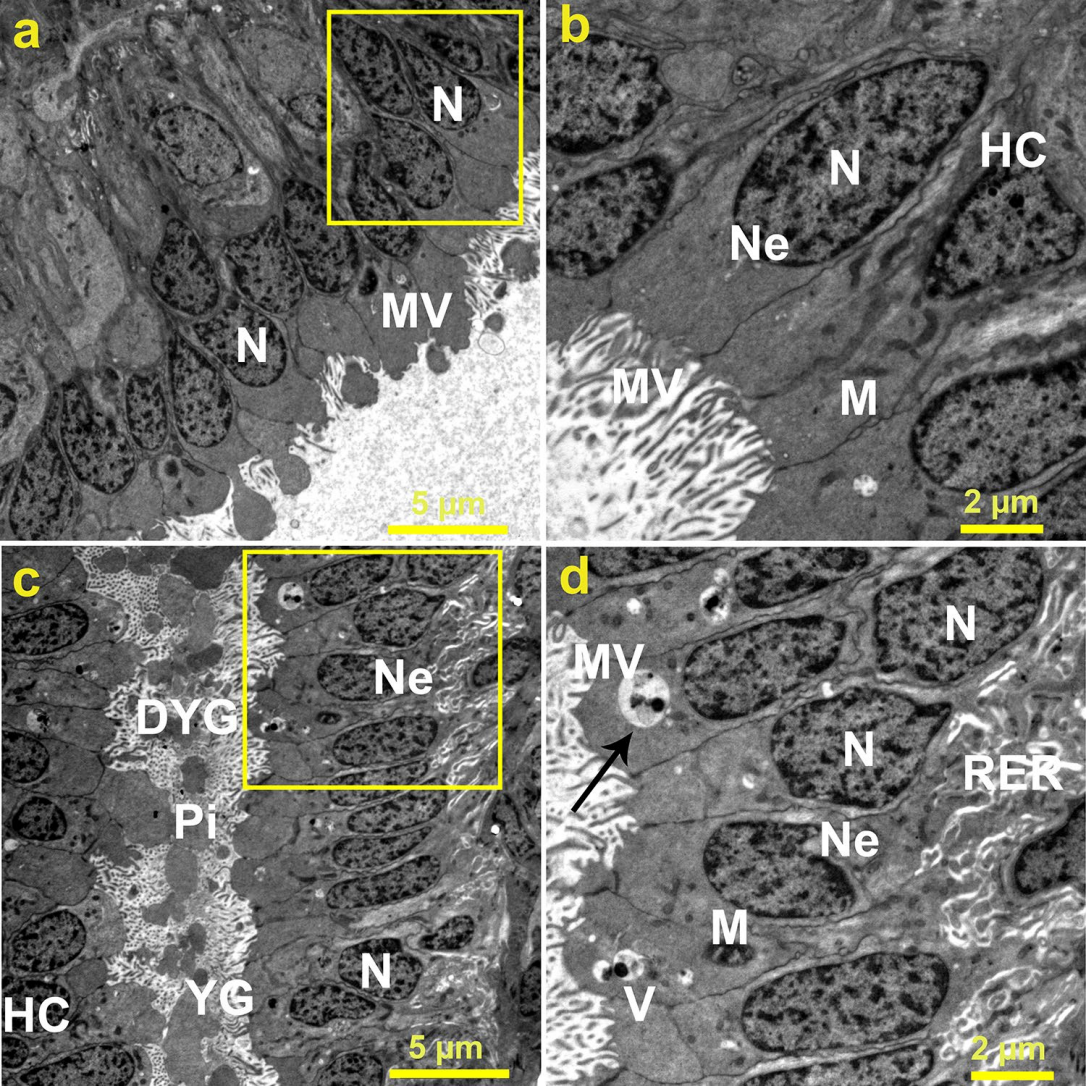
Transmission electron micrographs of (**a**) oocytes of *B. polychresta* from the control group shows normal nucleus, and thin layer of microvilli and (**b**) a magnified section of the oocytes of the control group reveals normal organelles, nucleus, microvilli, and heterochromatin. (**c**) exhibits oocytes of *B. polychresta* from Ag-NPs-treated group 3 with various irregular structures, involving follicular layer composed of elongated nuclei, microvilli, ooplasm region with small-sized yolk granules or with damaged yolk granules, and pinosomes and (**d**) a magnified section of the oocytes of the Ag-NPs-treated group shows elongated nucleus, vacuoles, rough endoplasmic reticulum, and electron dense particles. Nucleus (N), microvilli (MV), yolk granules (YG), damaged yolk granules (DYG), pinosomes (Pi), heterochromatin (HC), vacuoles (V), rough endoplasmic reticulum (RER), the arrow indicates electron dense particles, and the yellow squares point to the magnified sections. Three ovarian tissues from different beetles of Ag-NPs-treated group 3 and the control group were investigated by TEM.

Additionally, the ultrastructural changes of oocytes were also analyzed in the beetles after the application of Ag-NPs in comparison with the untreated group (Fig. 7). For the control group, the oocytes possessed normal structures, involving heterochromatic nuclei alongside standard envelopes for nuclei. Additionally, the oocytes were also characterized by the presence of regular cytoplasmic organelles, and follicular epithelial cells (FECs), which bounded the egg cell (oocyte) as shown in Figs. 6b and 7a. Moreover, the oocyte exhibited a standard structure of ooplasm, which incorporated mitochondria, yolk granules, and droplets of lipid. In the case of Ag-NPs-treated group 3, noticeable cellular disintegration of the oocyte could be perceived in the TEM micrographs. Furthermore, FECs were deformed and emerged with malformed nuclei associated with irregular nuclear envelop. In addition, fragmented and enlarged mitochondria as abnormal characterization could be identified (Fig. 7c,d) in addition to broadened rough endoplasmic reticulum and vacuolated cytoplasm as a consequence of Ag^+^ treatment. Furthermore, Fig. 7c reveals the presence of degenerated yolk granules. Table 1 summarizes the ultrastructural anomalies in the ovarian tissues of the beetles as a result of exposure to Ag-NPs. Moreover, quantifications of normal and malformed organelles in the ovarian tissues examined by TEM were estimated to highlight the substantial differences between the ovarian tissues of the control group and Ag-NPs-treated group 3 as represented in Table 2. Overall, the treatment of *B. polychresta* females with a single dose of 0.03 mg/g body weight of Ag-NPs significantly altered the ovarian structure of the beetles.

**Table 1:** Ultrastructural alterations in ovaries of *B. polychresta* as consequence of exposure to Ag-NPs.

Organelles	Ultrastructural alterations
Trophocytes	Malformation of Pyknotic nuclei Prominent disintegration of mitochondria Distinct degradation of interstitial cells
Oocytes	Noticeable cellular disintegration of the oocyte Deformation of the follicular epithelial cells (FECs) and irregular nuclei and nuclear envelop Fragmented and enlarged mitochondria Broadened rough endoplasmic reticulum and vacuolated cytoplasm Degenerated yolk granules

**Table 2:** Quantification of the organelles altered in ovaries of *B. polychresta* as a result of exposure to Ag-NPs.

Organelles	Control group (quantification and observation of organelles)	Ag-NPs-treated group 3 (quantification and observation of organelles)
Nucleus	82 Normal spherical nucleus	98 degenerated and abnormal elongated nucleus
Nuclear envelope	82 regular nuclear envelope	98 irregular nuclear envelope
Heterochromatin	Normal heterochromatin	36 Abnormal condensed heterochromatin
Mitochondria	240 Normal mitochondria	106 Malformed mitochondria

Quantifications were estimated from three ovarian tissues from different beetles of Ag-NPs-treated group 3 and the control group.

## Discussion

The comprehensive and growing applications of fabricated Ag-NPs are a cause of bio-burdens on account of the potential detrimental consequences to the environment or human beings. Given that the negative influences of Ag-NPs are still being questioned, recent studies have attempted to provide insights into their toxicity towards living organisms as well as monitor their environmental performance and routes^3^. However, since investigation of the toxicity of Ag-NPs in humans is unattainable and very restricted in mammals, the utilization of non-mammal model species is alternatively established^18,19^. These organisms are adopted due to their favorable traits, including a lack of specialized requisites for survival and a short-life cycle^20^. It has been reported that darkling or tenebrionid beetles (Coleoptera) are an advantageous examination model for the relevant studies owing to their diverse nature, and reasonable endurance to several treatments^21^. We thus used *B. polychresta* in this study, which is considered one of the important indicators of toxic substances.

Based on TEM analysis, the Ag-NPs had an average diameter of 21.5 ± 6.0 nm in the current study. The size of Ag-NPs is a crucial factor in determining whether they can penetrate biological membranes, enabling them to bind several internal molecules without altering the fundamental structure of the nanoparticle^2,3^. Previous research reported that 20 nm citrate-coated Ag-NPs instigated higher cellular toxicity than 110 nm Ag-NPs, which triggered severe inflammation in the lungs of mice^22^. This is likely due to the greater surface area to volume proportion since Ag-NPs with smaller possess a quicker rate of Ag^+^ emancipation in the surrounding environment, resulting in improved bioavailability, promoted distribution, and toxicity of Ag^+^ compared to larger Ag-NPs^23^. However, uncapped or capped Ag-NPs with various sizes up to 100 nm have been implemented for studying their toxicity in relation to different animals^3^. Furthermore, it is believed that the toxicity of Ag-NPs is closely linked to some paramount features, including size, concentration, agglomeration, modifications by chemical coating, and charge over their surface, as well as the synthesis procedures utilized^2^.

Studying the correlation between Ag-NPs dosage and mortality was accomplished for diverse organisms, including *Panagrellus redivivus*, *Folsomia candida*, and *Drosophila melanogaster*^24–26^. In the current research, using death as an endpoint, it could be deduced from the data that the LD50 was perceived at an Ag-NPs dose of 0.05 mg/g body weight, while the sublethal dose was 0.03 mg/g body weight after 30 days of injection. On the contrary, for wistar rats, LD50 was explored to be 5000 mg/kg^27^, which reflects the greater sensitivity of *B. polychresta* with regards to Ag^+^ toxicity. These findings are in line with the previous studies conducted on insects and other invertebrates as favorable models^3,26^.

It has been reported that the bioaccumulation of Ag-NPs in diverse organisms, such as mammals, nematodes, and insects provoked considerable alterations in different organs, including the reproductive system after treatment with the NPs (oral, intravenous, intraperitoneal or subcutaneous)^1^. An X-ray examination of ovaries harvested from *B. polychresta* revealed the presence of four abundant elements (C, P, S, and O) in the control group, although six elements were detected in the Ag-NPs-treated group 3, including (Ca, C, P, S, O, and Ag) as a consequence of the expected dissolution of Ag^+^ from Ag-NPs. The incidence of Ca element in treated group 3 is likely to be related to the calcium dysregulation on account of the expanded levels of ROS, which led to high oxidative stress^28^. Moreover, the accumulation of Ag^+^ in the midgut tissues of the Ag-NPs-treated group 3 and the crucial disturbance in other elements, including Ca corroborate the previous findings with regards to ovaries.

The MDA is the dominant parameter for estimating the peroxidation of lipids^29,30^. Our findings revealed extreme augmentation of the MDA level, and due to the great toxicity of MDA, it could be presumed that the high prospective interaction between DNA and protein with MDA impaired these molecules. This attitude clearly implies substantial genotoxicity in *B. polychresta from* Ag-NPs treated group 3, which could be evidenced by comet analysis and flow cytometry. This exposition matches those observed in the previous studies carried out on Wistar rats subjected to zinc oxide nanoparticles, in which their findings corroborated the close link between DNA damage and MDA level^31^. In the same manner, the activities of AST and ALT were significantly heightened in the case of Ag-NPs treated group 3 in comparison with the control group. Although the functions of these enzymes are not fully identified in beetles, they allude to the immunity and pathological alterations of insects^32^. Correspondingly, previous findings demonstrated the increase in the activity of ALT in *Bombyx mori* as a result of the administration of toxic compounds^33^.

The toxic and genotoxic consequences of nanomaterials are thought to be modulated by antioxidant enzymes^2,34^. In contrast, compared with the Ag-NP-untreated control group, total protein content, and antioxidant enzymes, including CAT, GPx, GST, and SOD levels were substantially lessened in the ovary homogenates of the Ag-NPs treated group 3. This could be attributed to the deterioration of the antioxidant defense system due to the extreme generation of ROS, which gave rise to the devastation of lipids, DNA, and proteins, and further enhances apoptosis of cells^35,36^. Moreover, Nair and Choi concluded that industrial Ag-NPs influenced the gene expression of glutathione S-transferase (GST) in the aquatic *Chironomus riparius* (Meigen)^37^. According to Mao et al.^38^, Ag-NPs at sublethal doses induced mortality and had adverse impacts on the growth of *Drosophila melanogaster*, and led to the aggregation of ROS in the fly tissues of *D. melanogaster*, resulting in ROS-mediated apoptosis, autolysis, and autophagy. A recent report revealed the antifungal competency of copper nanoparticles (Cu-NPs) against *Fusarium kuroshium*, manifesting the significant enrichment of the oxidation–reduction processes and enzymes with oxidoreductase activity. Moreover, *F. kuroshium* attempted to hinder the shuttle and interactions of Cu^+^ with some organelles, such as Golgi bodies and the mitochondria^39^. Another study reported the capacity of magnesium oxide nanoparticles (Mg-NPs) to thwart the growth of Gram-negative and Gram-positive bacteria by generating high amounts of ROS as a dominant mechanism. They presumed that the underlying cause behind the uncontrolled ROS generation is the release of Mg^2^^+^ ions from the nanoparticle, which leads to extreme oxidative stress. This provokes a critical lack of proteins, carbohydrates, and lipids, resulting in bacterial cell death^40^.

It has been described that the high toxicity of Ag-NPs in vitro in relation to different types of cells at levels of 5–10 µg/ml and sizes within a range of 10–100 nm could frustrate the function of mitochondria^2^. From previous investigations, it is presumed that the capability of Ag-NPs to invade the cells towards the mitochondria brings about oxidative stress^2,3,23^. Accordingly, prior research postulated that the cellular toxicity of Ag-NPs was predominantly incited through disturbing the mitochondrial mechanism by disordering the GSH mediated antioxidant scavenge system, promoting peroxidation of lipids, and the genes responsible for ROS, triggering damage to DNA, apoptosis, and necrosis^41^. In addition to these in vitro impacts, as a result of in vivo investigations, Ag-NPs engendered negative influences on reproduction, and even led to malformations in diverse non-mammalian model organisms^42^. Our findings support these concepts since the group treated with Ag-NPs exposed a higher ratio of apoptotic cells and a lower rate of viable cells in comparison with the control group. These results are in complete agreement with the findings of Bharani and Namasivayam^43^, who perceived the manifest effects of Ag-NPs on apoptotic processes of Lepidoptera: Noctuidae, *Spodoptera litura* (Fab.). It is worth mentioning that the adverse effects of Ag-NPs on the cell cycle could stem from the stimulation of DNA hypermethylation, which might further affect the cells at an epigenomic level^44^. Consistently, we found that the level of DNA damage was significantly higher in the Ag- NPs-exposed group than in the unexposed group. The ovarian tissues, which were not directly exposed to Ag-NPs, were used to quantify DNA injury. This confirms that Ag-NPs penetrate the ovarian cell membranes and interact with intracellular targets. In accordance with the present results, Nichols et al.^45^ evinced the growth in DNA damage rate owing to the toxicity of cerium dioxide (CeO_2_), silicon dioxide (SiO_2_), and titanium dioxide nanoparticles (TiO_2_).

Moreover, it has been reported that high oxidative DNA damage in workers subjected to zinc oxide nanoparticles is due to overflow of ROS, which could give rise to cytotoxicity by inflammatory responses in addition to mitochondrial membrane alterations^46,47^.

**Figure 8:**
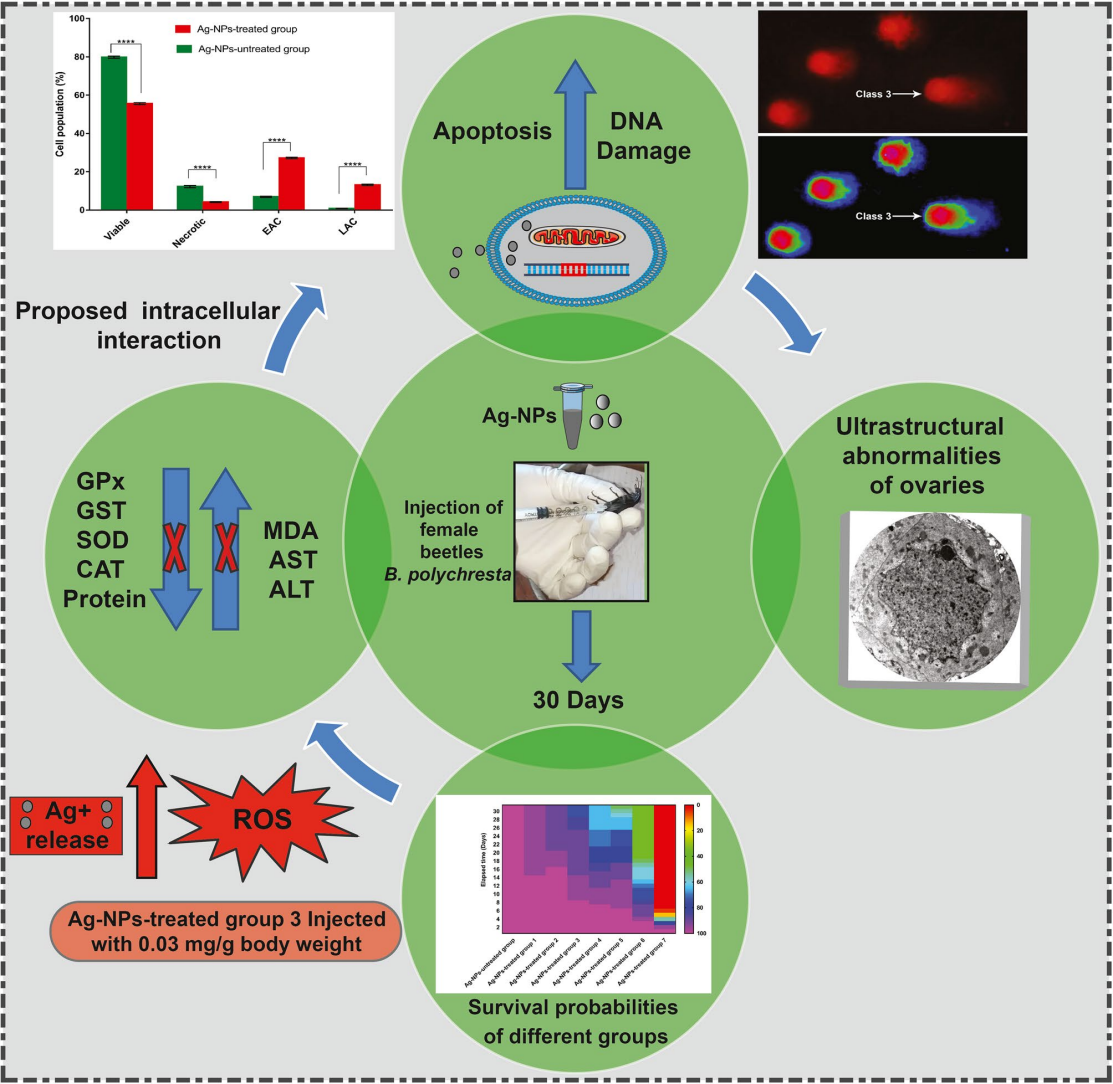
Schematic illustration reveals the physiological and pathophysiological alterations in the ovarian tissues of darkling beetles as a consequence of deleterious influence of Ag-NPs.

Fertility, reproduction, and embryonic growth are crucial to a species' survival, emphasizing the importance of increasing public understanding of the toxicity of NPs on the reproductive system^1^. Similarly, NPs pose a possible danger to the vulnerable female population, and their toxicity has been studied in various female reproductive health models^48^. In the current research, various histological and ultrastructural abnormalities were discerned in the ovarian tissues of *B. polychresta* treated with Ag-NPs. These abnormalities include the involvement of apoptotic cells, lytic regions in the cytoplasm, and disintegrated yolk granules. The emergence of apoptotic cells verifies the findings of the comet assay. Our findings are consistent with those of El-Ashram et al.^16^, who found an abnormality in the oogenesis with Au exposure in *Trachyderma hispida*. Nuclear deformities are the first symptoms of cell death^49^, which are evident in the trophocytes in this study. In addition, toxicity of Ag-NPs stimulates lysosomal hydrolase, which is formed in vacuolated areas of the cytoplasm in addition to other abnormalities in the ovarian cells. Moreover, we found out trophocytes with pyknotic nuclei associated with damage to mitochondrial integrity, as well as certain interstitial fibroblastic epithelial cells. Kheirallah et al.^17^ reported comparable deformities in the ovarian cells of beetles administered with NiO-NPs at the respective sublethal dose. Besides, nuclear and cytoplasmic abnormalities were perceived in the follicular epithelial cells (FECs). It is believed that the toxicity of Ag-NPs varies in relation to targeted organisms due to the difference in the defense mechanisms used to eradicate heavy metals. We presume that the small size of Ag-NPs facilitates their transfer through different biological membranes to bind with target organelles. Predominantly, intact Ag-NPs and Ag^+^ discharged from the nanoparticles are believed to be detrimental by inspiring the damage of membrane integrity, overproduction or aberration of ROS, oxidation of proteins and altering their conformations, leading to their dysfunction, malfunction of mitochondria, DNA damage, and impediment of cell propagation^23,50^. Several approaches have been implemented in order to appraise the toxicity of Ag-NPs in studied model organisms. Previous studies in rats and rabbits exposed to nanoparticles (oral, intravenous, intraperitoneal, or subcutaneous) revealed changes in mammalian organs linked with reproductive and even development progressions, which could be attributed to oxidative stress and inflammation as a result of ROS production as the leading mediating cause of cellular toxicity^1^. Our assumptions are in complete agreement with previous reports conducted on different mammals^3,51^. Altogether, Fig. 8 concludes the potential biological mechanisms in the ovarian tissues of darkling beetles as a consequence of deleterious influence of Ag-NPs. Furthermore, our findings clearly exhibit the potential utilization of darkling beetles as bio-indicators for the toxicity of diverse nanoparticles.

## Conclusion

In the current study, we assessed the toxicity of Ag-NPs at the lowest level on the ovaries of Egyptian female darkling beetles (*B. polychresta)*. The exposure to a single application of Ag-NPs resulted in substantial dysfunctions in several physiological properties of the treated insects. Specifically, critical interruptions were discerned in the antioxidant defense system associated with substantial growth in apoptotic cells and genotoxicity for the Ag-NPs treated group in comparison with the control insects. Besides, ultrastructural changes were observed in the ovarian tissues of the darkling beetles instigated by Ag-NP exposure. These considerable alterations are most likely related to the liberation of Ag^+^ from Ag-NPs, which can penetrate different biological barriers and bind to mitochondria. Altogether, the findings reported in this study are striking since they strongly boost the capability to extrapolate the impacts of Ag-NPs on large organisms along with the ecosystem.

## Materials and methods

### Samples collection and identification

The garden of the Faculty of Science, Alexandria University, Elshatby, Alexandria, Egypt (latitude: 31.207189°N and longitude: 29.919110°E) was identified as an unpolluted area^17^; thus, it was chosen for beetle collection. A total of 320 beetles were picked up and instantly transferred to the lab. The beetles were then identified as *B. polychresta*, Family: Tenebrionidae, at the Entomology Department, Faculty of Agriculture, Alexandria University prior to being maintained in cages containing natural vegetation and soil collected from the collection region. The humidity and temperature were adapted to be consistent with their native habitat at 85% RH and 29.3 °C, respectively. Females were discernable according to the 8th sternite median cleft, which is shorter in females than in males (Fig. S3), and the mean body weight of adult female beetles was 1.89 g. After 5 days of acclimatization, a total of 160 females were randomly categorized into eight groups, and each one included 20 adult females. Therefore, a control group, and seven groups injected with different concentrations of Ag-NPs were established.

### Characterizations of Ag-NPs

Ag-NPs supplied by Sigma-Aldrich Co. (St. Louis MO) were characterized by means of a transmission electron microscope (JEOL, JEM-2100 plus Electron Microscope, Japan) at an accelerating voltage of 200 kV.

### Ag-NPs exposure

A stock solution of Ag-NPs suspension with a final concentration of 0.4 mg/ml was prepared in a saline solution before sonication for 30 min in a water bath sonicator to avoid the aggregation of Ag-NPs. To determine the sublethal dose, adult females were injected with a single dose of seven diluted Ag-NPs solutions (0.01, 0.02, 0.03, 0.04, 0.05, 0.06, and 0.07 mg/g body weight) under sterile conditions. Concurrently, the Ag-NP-untreated control group was injected with an equivalent amount of normal saline solution. The beetles were injected laterally between the 4th and 5th abdominal segments using one ml BD hypodermic syringe (27G, 1"2/ needle) as depicted in Fig. S4. Following this, the survival of the specimens was observed daily for 30 days. Accordingly, apoptotic, genotoxicity, biochemical, and ultrastructural alterations were investigated in Ag-NPs-treated group 3 that was injected with a sublethal dose of 0.03 mg/g body weight in comparison with the control group.

### Ag-NPs bioaccumulation in ovarian tissues of *B. polychresta*

The content of Ag^+^ in ovaries of Ag-NPs-treated group 3 was evaluated employing a scanning electron microscope (Jeol JSM-5300, Japan) equipped with a Link-Isis energy dispersive X-ray micro- analyzer (EDX). For 110 s, a static location (X500) was randomly estimated. For this analysis, we used nine specimens of ovarian and midgut tissues from Ag-NPs treated beetles and the control group in order to explore the correlation between the accumulation of Ag-NPs and the injection site. The SEM–EDX program automatically allocated the identity of each peak. The intensity of each element in the analyzed sample was compared to the comparable element in calibration standards. As this study aims to assess the toxicity of Ag-NPs in relation to ovaries in females, the ovarian tissues from Ag-NPs-treated group 3 and the control group were used for further investigations.

### Biochemical analyses

To estimate the activities of enzymes and protein content, ovaries from the survived beetles of Ag-NPs-treated group 3 and the control group were harvested, dissected on ice, and then washed twice with a saline solution prior to being carefully blotted using filter paper. After weighing the ovaries, they were homogenized in a 67 mM potassium phosphate solution (pH 7) with a tissue to buffer ratio of 1:5, respectively. The homogenized ovaries were clarified by centrifugation at 10,000 xg and 4 °C for 30 min, and the supernatants were then obtained for conducting the biochemical assays. The protein content was estimated in accordance with Bradford’s method. The activities of aspartate aminotransferase (AST), and alanine aminotransferase (ALT) were determined using Tietz's method^52^. Using the respective kits supplied by Cayman Chemical Company (Michigan, USA), Catalase activity (CAT), Glutathione S-transferase level (GST), and the level of malondialdehyde (MDA) in the homogenized ovaries were measured according to the manufacturers' instructions. Besides, the activity of glutathione peroxidase (GPx) was determined following Paglia and Valentines' method^53^.

### Assessment of DNA damage using comet assay

The comet assay was adopted to assess DNA damage in alkaline conditions, adapted from a previously described approach^54^. In order to prepare a suspension of ovarian cells, the ovarian tissues were gently soaked in cold Hanks' balanced salt solution comprising 20 mM ethylenediaminetetraacetic acid (EDTA) and 10% dimethyl sulfoxide (DMSO). Next, the slides of the Star Frost microscope were mounted with 0.65% normal-melting agarose. The cell suspension was then blended with a final concentration of 0.65% low-melting agarose solution and distributed over slides. Following solidification of agarose at 4 °C for 20 min, the slides were overlaid with 120 ml of 0.65% low-melting agarose. Subsequently, to induce the lysis of cell membranes, the slides were submerged in the respective lysis buffer overnight at 4 °C. Afterwards, the slides were thoroughly washed using milli-Q water, followed by electrophoresis at 300 mA for 25 min. Next, the slides were washed with 0.4 M tris pH 7.5 for 15 min (3 times with 5 min for each) before being dehydrated in 96% ethanol for 35 s. To probe the comet, the slides were rehydrated using milli-Q water, followed by staining with ethidium bromide solution (20 mg/ml). The slides were investigated by means of a Leica DMLS microscope (400 × magnification) to perceive the different classes of comet cells. For Ag-NPs-treated group 3 and the control group, five slides were developed and 100 comets per slide were inspected. The images of comet assay were further analyzed using ImageJ Fiji software (https://imagej.net/software/fiji/downloads).

### Cell viability assay by flow cytometry

The viability of cells was appraised employing the annexin V-FITC kit (Sigma–Aldrich, Germany) in accordance with the manufacturers' procedures. Female beetles were anesthetized and the ovaries were dissected on ice, followed by homogenization of ovarian tissues in cold phosphate buffer saline (PBS, pH 7.4) at 4 °C to collect the cell suspensions in the supernatants. The cells were harvested and washed three times with PBS prior to being resuspended in 195 μl binding buffer. Following this, the cell suspensions were blended with 5 μL of annexin V-FITC conjugate reagent and maintained for 10 min under dark conditions. After harvesting and washing of cells, the resuspended cells in 190 μl binding buffer were blended with 10 μl propidium iodide solution. Annexin V-FITC/PI stained cells were instantly examined using a flow cytometry analyzer (Becton Dickinson, Franklin Lakes, NJ, USA) as previously proposed^54^. The obtained data was analyzed using CellQuest Pro software version 5.2.1, 2005 (BD Biosciences, San Jose, CA).

### Ultrastructural investigation of the ovaries from *B. polychresta*

The ovaries were separated by forceps from the genital system and then fixed in 4F1G buffer (pH 7.2) containing 40% formaldehyde (10 ml), 50% glutaraldehyde (2 ml), monobasic sodium phosphate (1.16 mg), NaOH (0.27 mg), and ultrapure water (up to 100 ml). Afterwards, the ovaries were washed in PBS (pH 7.4) for 3 h at 4 °C, followed by fixation for 2 h in 2% OsO_4_ prepared in PBS. Subsequently, the tissues were dehydrated using different ethanol gradients before being embedded in an Epon-Araldite mixture. The tissues were then sectioned into ultrathin sections of 70 nm on 200 mesh naked copper grids. After that, the ultrathin sections were visualized by staining with uranyl acetate and lead citrate before being surveyed employing a transmission electron microscope (TEM, Joel 100 CX, Japan) at an acceleration voltage of 80 kV at the Faculty of Science, Alexandria University.

### Statistical analysis

The particle size of Ag-NPs and the mortality of different group of beetles after exposure to Ag-NPs were analyzed using Origin software (V. 8) and Statistica software (V. 8), respectively. All investigations were carried out from 3 to 6 independent experiments, and the data were normalized before analysis adopting a Student's *t*-test using Mann Whitney test in GraphPad Prism software (V. 7). The values are expressed as mean ± SEM and the significant results were considered at *P* ≤ 0.05, whereas the high significant values were considered at *P* ≤ 0.01, *P* ≤ 0.001, and *P* ≤ 0.0001.

## Supplementary Information

Supplementary Information.

## Data Availability

The datasets used and/or analyzed during the current study are available from the corresponding authors on reasonable request.

## References

[1] Ema M, Okuda H, Gamo M, Honda K (2017). A review of reproductive and developmental toxicity of silver nanoparticles in laboratory animals. Reprod. Toxicol..

[2] Akter M (2018). A systematic review on silver nanoparticles-induced cytotoxicity: Physicochemical properties and perspectives. J. Adv. Res..

[3] Tortella GR (2020). Silver nanoparticles: Toxicity in model organisms as an overview of its hazard for human health and the environment. J. Hazard. Mater..

[4] Chandrasekharan S, Chinnasamy G, Bhatnagar S (2022). Sustainable phyto-fabrication of silver nanoparticles using *Gmelina arborea* exhibit antimicrobial and biofilm inhibition activity. Sci. Rep..

[5] Shukla G, Gaurav SS, Singh A, Rani P (2022). Synthesis of mycogenic silver nanoparticles by Fusarium pallidoroseum and evaluation of its larvicidal effect against white grubs (Holotrichia sp.). Mater. Today Proc..

[6] Calderón-Jiménez B (2017). Silver Nanoparticles: Technological Advances, Societal Impacts, and Metrological Challenges. Front Chem.

[7] Pulit-Prociak J, Banach M (2016). Silver nanoparticles—A material of the future…?. Open Chem..

[8] Beer C, Foldbjerg R, Hayashi Y, Sutherland DS, Autrup H (2012). Toxicity of silver nanoparticles—Nanoparticle or silver ion?. Toxicol. Lett..

[9] Chernousova S, Epple M (2013). Silver as Antibacterial Agent: Ion, Nanoparticle, and Metal. Angew. Chem. Int. Ed..

[10] Tang J (2010). Silver nanoparticles crossing through and distribution in the blood-brain barrier in vitro. J. Nanosci. Nanotechnol..

[11] Wisniewski P (2015). Adult exposure to bisphenol A (BPA) in Wistar rats reduces sperm quality with disruption of the hypothalamic–pituitary–testicular axis. Toxicology.

[12] Charehsaz M (2016). Effects of developmental exposure to silver in ionic and nanoparticle form: a study in rats. Daru J. Fac. Pharm..

[13] Melnik EA (2013). Transfer of Silver Nanoparticles through the Placenta and Breast Milk during in vivo Experiments on Rats. Acta Nat..

[14] Wu J (2015). Effects of prenatal exposure to silver nanoparticles on spatial cognition and hippocampal neurodevelopment in rats. Environ. Res..

[15] Elnoury MAH (2013). Study of the effects of silver nanoparticles exposure on the ovary of rats. Life Sci. J..

[16] El-Ashram S, Kheirallah DAM, El-Samad LM, Toto NA (2020). Relative expression of microRNAs, apoptosis, and ultrastructure anomalies induced by gold nanoparticles in Trachyderma hispida (Coleoptera: Tenebrionidae). PLoS ONE.

[17] Kheirallah DAM, El-Samad LM, Abdel-Moneim AM (2021). DNA damage and ovarian ultrastructural lesions induced by nickel oxide nano-particles in Blaps polycresta (Coleoptera: Tenebrionidae). Sci. Total Environ..

[18] Benelli G (2018). Mode of action of nanoparticles against insects. Environ. Sci. Pollut. Res..

[19] Moon J, Kwak JI, An Y-J (2019). The effects of silver nanomaterial shape and size on toxicity to Caenorhabditis elegans in soil media. Chemosphere.

[20] Kheirallah D, El-Samad L (2019). Spermatogenic alterations in the ground beetle Trachyderma hispida (Coleoptera: Tenebrionidae) induced by ceramic industrial pollution. African Entomol..

[21] Osman W, Shonouda M (2017). X-ray metal assessment and ovarian ultrastructure alterations of the beetle, Blaps polycresta (Coleoptera, Tenebrionidae), inhabiting polluted soil. Environ. Sci. Pollut. Res. Int..

[22] Wang X (2014). Use of coated silver nanoparticles to understand the relationship of particle dissolution and bioavailability to cell and lung toxicological potential. Small.

[23] Ferdous Z, Nemmar A (2020). Health impact of silver nanoparticles: a review of the biodistribution and toxicity following various routes of exposure. Int. J. Mol. Sci..

[24] Mahmoud WM, Abdelmoneim TS, Elazzazy AM (2016). The impact of silver nanoparticles produced by bacillus pumilus as antimicrobial and nematicide. Front. Microbiol..

[25] McKee MS (2017). Collembola reproduction decreases with aging of silver nanoparticles in a sewage sludge-treated soil. Front. Environ. Sci..

[26] Ávalos A, Haza AI, Drosopoulou E, Mavragani-Tsipidou P, Morales P (2015). In vivo genotoxicity assesment of silver nanoparticles of different sizes by the Somatic Mutation and Recombination Test (SMART) on Drosophila. Food Chem. Toxicol..

[27] Adeyemi OS, Adewumi I (2014). Biochemical evaluation of silver nanoparticles in wistar rats. Int. Scholarly Res. Notices.

[28] Ziemińska E, Stafiej A, Strużyńska L (2014). The role of the glutamatergic NMDA receptor in nanosilver-evoked neurotoxicity in primary cultures of cerebellar granule cells. Toxicology.

[29] CEPOI L, Ludmila RUDI, CHIRIAC T, MISCU V, RUDIC V (2020). Malondialdehyde-a potential marker of nanoparticle toxicity in an aquatic environment. One Health Risk Manag..

[30] Ramzan M (2021). Mitigation of bacterial spot disease induced biotic stress in *Capsicum annuum* L. cultivars via antioxidant enzymes and isoforms. Sci. Rep..

[31] Husain W, Araak J, Ibrahim O (2019). Effect of zinc oxide nanoparticles on sperm cell comet assay, testis malondialdehyde and glutathione peroxidase levels in adult rats. Online J. Vet. Res..

[32] Łoś A, Strachecka A (2018). Fast and cost-effective biochemical spectrophotometric analysis of solution of insect "Blood" and body surface elution. Sensors (Basel).

[33] Inagaki Y, Matsumoto Y, Kataoka K, Matsuhashi N, Sekimizu K (2012). Evaluation of drug-induced tissue injury by measuring alanine aminotransferase (ALT) activity in silkworm hemolymph. BMC Pharmacol. Toxicol..

[34] Ram AK, Mallik M, Reddy RR, Suryawanshi AR, Alone PV (2022). Altered proteome in translation initiation fidelity defective eIF5G31R mutant causes oxidative stress and DNA damage. Sci. Rep..

[35] Miethling-Graff R (2014). Exposure to silver nanoparticles induces size- and dose-dependent oxidative stress and cytotoxicity in human colon carcinoma cells. Toxicol. In Vitro.

[36] Sriram MI, Kalishwaralal K, Barathmanikanth S, Gurunathani S (2012). Size-based cytotoxicity of silver nanoparticles in bovine retinal endothelial cells. Nanosci. Methods.

[37] Nair PMG, Park SY, Choi J (2013). Evaluation of the effect of silver nanoparticles and silver ions using stress responsive gene expression in *Chironomus riparius*. Chemosphere.

[38] Mao B-H, Chen Z-Y, Wang Y-J, Yan S-J (2018). Silver nanoparticles have lethal and sublethal adverse effects on development and longevity by inducing ROS-mediated stress responses. Sci. Rep..

[39] Ibarra-Laclette E (2022). Antifungal effect of copper nanoparticles against *Fusarium kuroshium*, an obligate symbiont of *Euwallacea kuroshio* ambrosia beetle. J. Fungi.

[40] Bhattacharya P, Dey A, Neogi S (2021). An insight into the mechanism of antibacterial activity by magnesium oxide nanoparticles. J. Mater. Chem. B.

[41] Haase A (2012). Effects of silver nanoparticles on primary mixed neural cell cultures: Uptake, oxidative stress and acute calcium responses. Toxicol. Sci..

[42] Zhang X-F, Gurunathan S, Kim J-H (2015). Effects of silver nanoparticles on neonatal testis development in mice. Int. J. Nanomed..

[43] Bharani RSA, Namasivayam SKR (2017). Biogenic silver nanoparticles mediated stress on developmental period and gut physiology of major lepidopteran pest Spodoptera litura (Fab.) (Lepidoptera: Noctuidae)—An eco-friendly approach of insect pest control. J. Environ. Chem. Eng..

[44] Mytych J, Zebrowski J, Lewinska A, Wnuk M (2017). Prolonged effects of silver nanoparticles on p53/p21 pathway-mediated proliferation, DNA damage response, and methylation parameters in HT22 hippocampal neuronal cells. Mol. Neurobiol..

[45] Nichols CE (2018). Reactive oxygen species damage drives cardiac and mitochondrial dysfunction following acute nano-titanium dioxide inhalation exposure. Nanotoxicology.

[46] Hou J (2018). Toxic effects of different types of zinc oxide nanoparticles on algae, plants, invertebrates, vertebrates and microorganisms. Chemosphere.

[47] Vimercati L (2020). Nanoparticles: An experimental study of zinc nanoparticles toxicity on marine crustaceans. General Overview on the Health Implications in Humans. Front. Publ. Health.

[48] Brohi RD (2017). Toxicity of nanoparticles on the reproductive system in animal models: A review. Front Pharmacol.

[49] Armenti AE, Zama AM, Passantino L, Uzumcu M (2008). Developmental methoxychlor exposure affects multiple reproductive parameters and ovarian folliculogenesis and gene expression in adult rats. Toxicol. Appl. Pharmacol..

[50] Zhang T, Wang L, Chen Q, Chen C (2014). Cytotoxic potential of silver nanoparticles. Yonsei Med. J..

[51] McShan D, Ray PC, Yu H (2014). Molecular toxicity mechanism of nanosilver. J. Food Drug Anal..

[52] Huang X-J (2006). Aspartate aminotransferase (AST/GOT) and alanine aminotransferase (ALT/GPT) detection techniques. Sensors (Basel).

[53] Cichoski AJ, Rotta RB, Scheuermann G, Cunha Junior A, Barin JS (2012). Investigation of glutathione peroxidase activity in chicken meat under different experimental conditions. Food Sci. Technol..

[54] El-Samad LM (2022). Time-delayed effects of a single application of AgNPs on structure of testes and functions in Blaps polychresta Forskal, 1775 (Coleoptera: Tenebrionidae). Sci. Total Environ..

