# Supplementary material for: Insights into Ag-NPs-mediated pathophysiology and ultrastructural aberrations in ovarian tissues of darkling beetles
Source: Sci Rep. 2022 Aug 16;12:13899. doi: 10.1038/s41598-022-17712-z (PMC9381597; doi:10.1038/s41598-022-17712-z)
Supplement: Supplementary file 1 — Supplementary Information. [file 41598_2022_17712_MOESM1_ESM.docx]

**Supplementary information**

**Insights into Ag-NPs-mediated pathophysiology and ultrastructural aberrations in ovarian tissues of darkling beetles**

Lamia M. El-Samad^1†*^, Mohamed A. Hassan^2†*^, Nahed R. Bakr^3^, Saeed El-Ashram^4,5^, Eman H. Radwan^3^, Karoline K. Abdul Aziz^3^, Hussein K. Hussein^1^, Abeer El Wakil^6^

^1^Department of Zoology, Faculty of Science, Alexandria University, Alexandria, Egypt;

^2^Protein Research Department, Genetic Engineering and Biotechnology Research Institute (GEBRI), City of Scientific Research and Technological Applications (SRTA-City), New Borg El-Arab City, P.O. Box: 21934 Alexandria, Egypt;

^3^Department of Zoology, Faculty of Science, Damanhour University, Egypt;

^4^College of Life Science and Engineering, Foshan University, 18 Jiangwan Street, Foshan, 528231, Guangdong Province, China;

^5^Faculty of Science, Kafrelsheikh University, Kafr El-Sheikh, 33516, Egypt;

^6^Department of Biological and Geological Sciences, Faculty of Education, Alexandria University, Egypt.

^†^These authors contributed equally to this study.

*Corresponding to: Lamia M. El-Samad E-mail: lamya.moustafa@alexu.edu.eg;

Mohamed A. Hassan E-mail: madel@srtacity.sci.eg

**Running title: Ovarian tissues of darkling beetles for monitoring Ag-NPs exposure.**


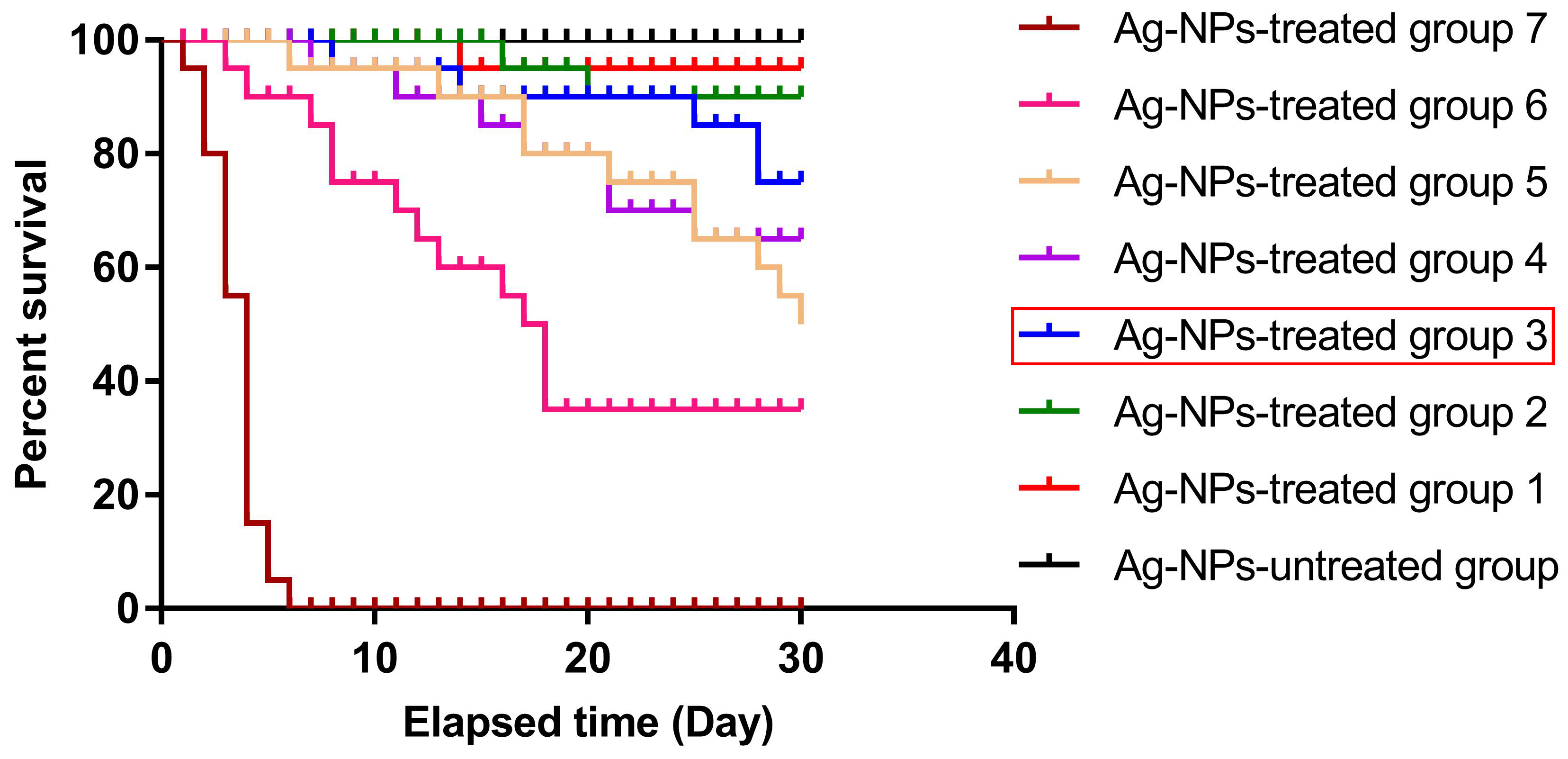


**Figure S1.** Kaplan–Meier survival analysis exhibits the survival probabilities of different groups from 1 to 7 of female beetles, B. polychresta, throughout 30 days after injection with Ag-NPs at various doses of 0.01, 0.02, 0.03, 0.04, 0.05, 0.06, and 0.07 mg/g body weight for each group, respectively. The red rectangle indicates the selected group for further investigations compared to the control group.


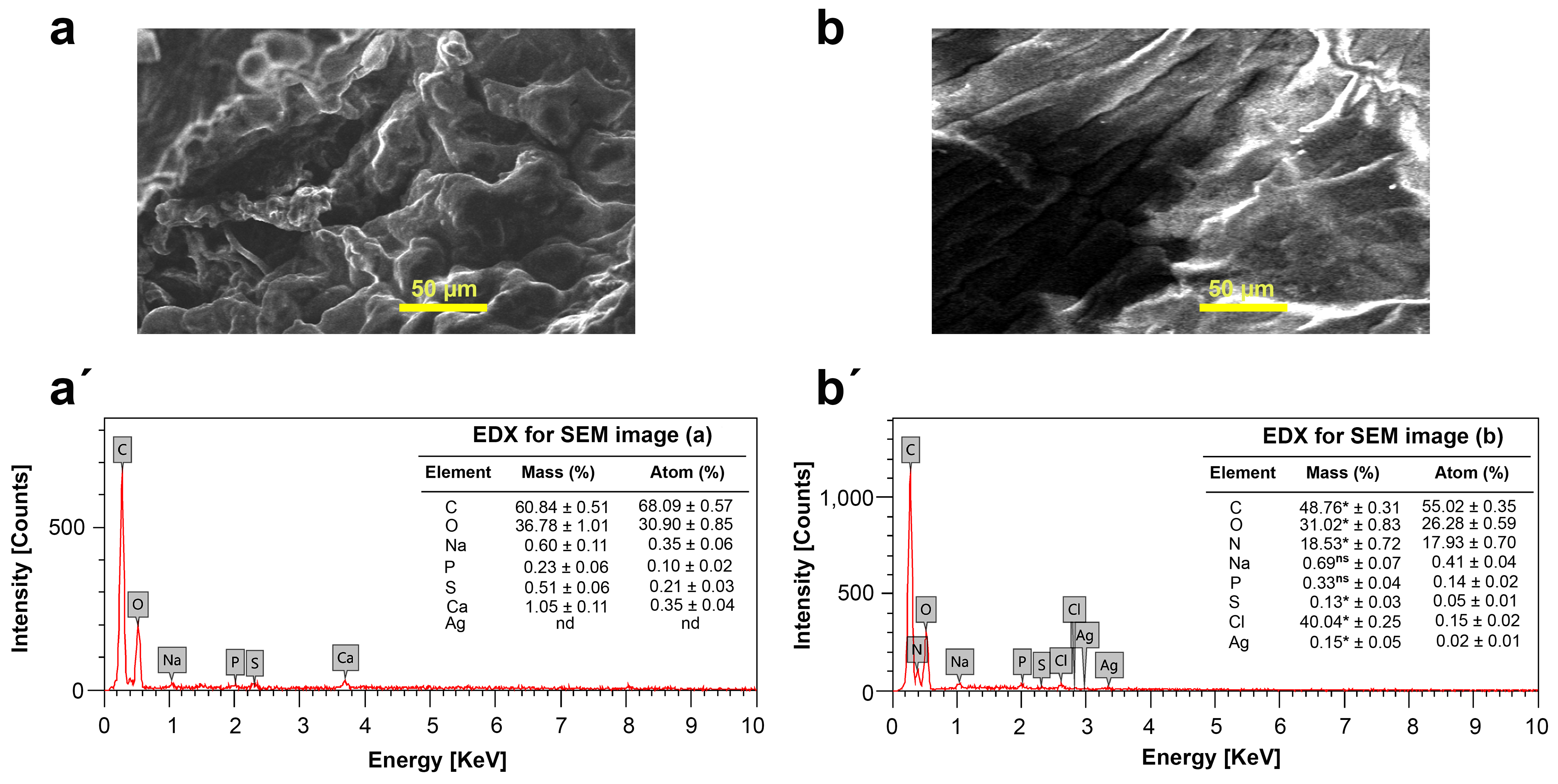


**Figure S2.** SEM of (a) midgut tissues from the control group and (b) midgut tissues from Ag-NPs-treated group 3. (a´) and (b´) EDX spectra of midgut tissues harvested from the control and Ag-NPs-treated group, respectively, exhibit quantitative analyses of the elements, signifying the presence of Ag^+^ in the Ag-NPs-treated group in comparison to the control beetles. The analysis was carried out using three sections of midgut tissues dissected from three beetles and the results are presented as mean ± SEM (**P* ≤ 0.05, and (ns) points to a non-significant difference).

**
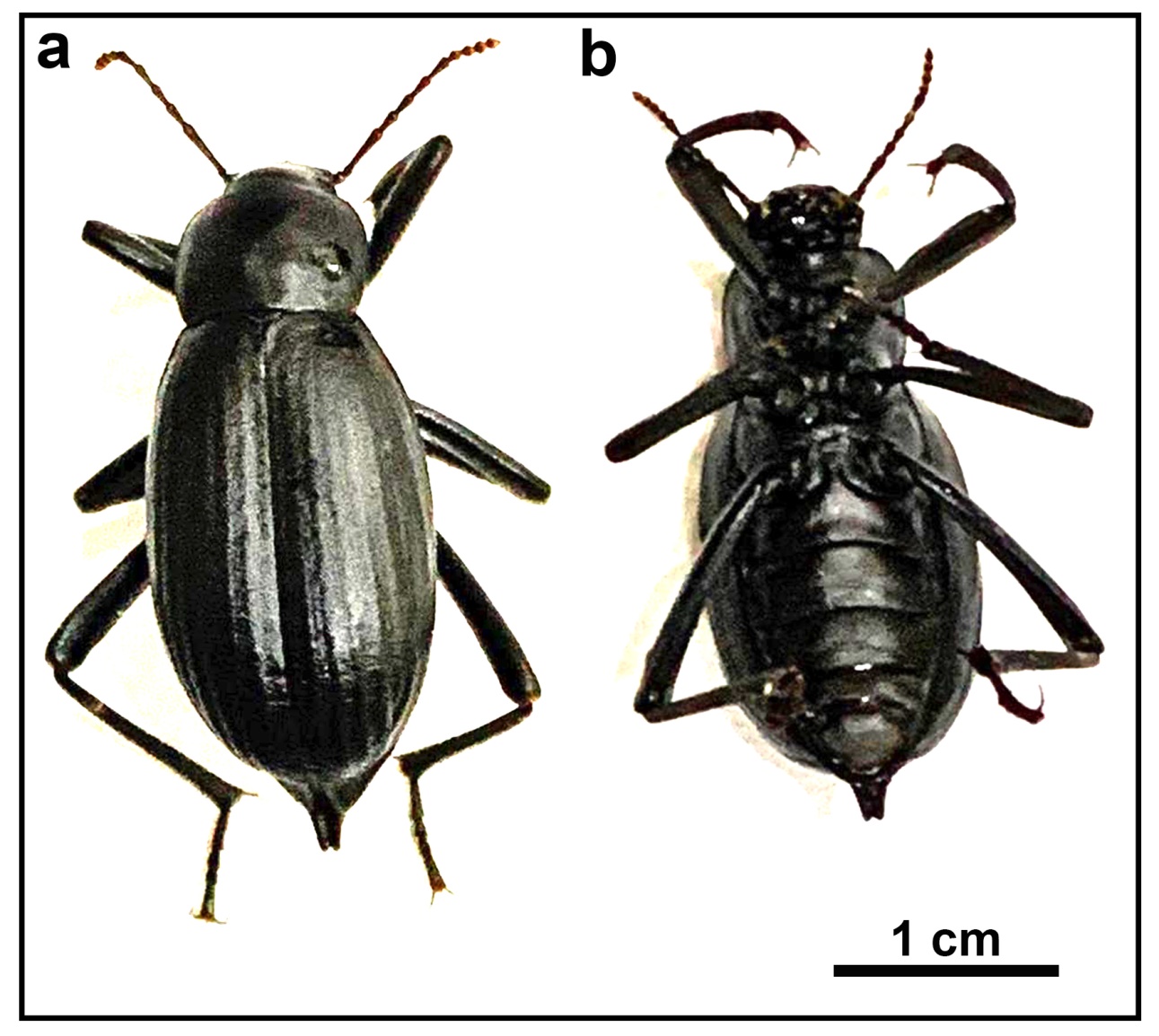
**

**Figure S3.** Dorsal (a) and ventral (b) views of female beetles (*B. polychresta*).

**
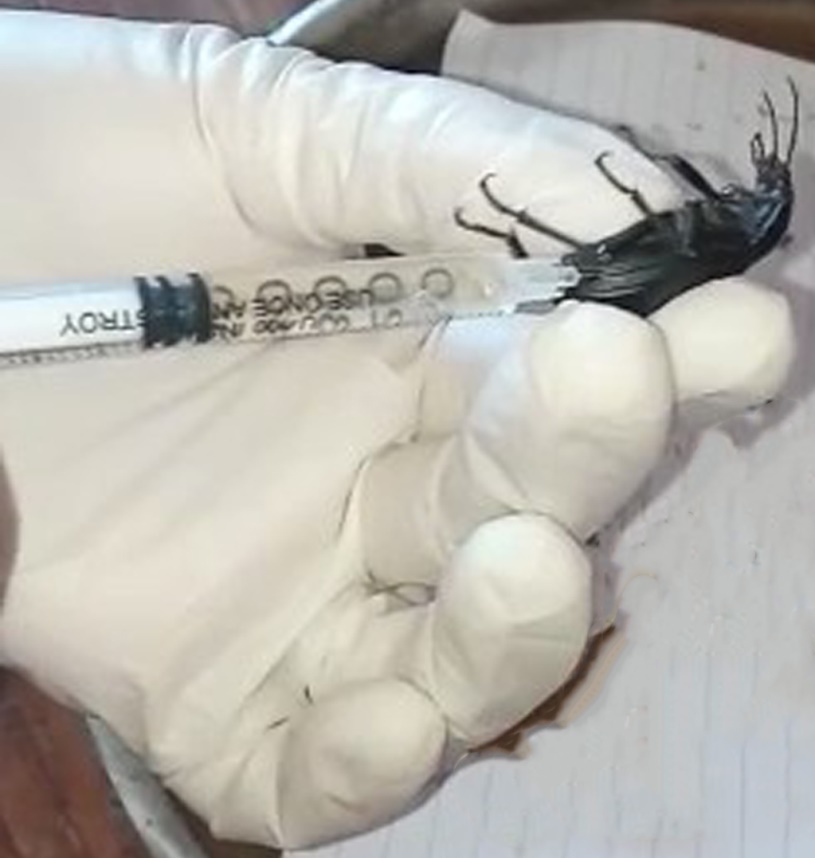
**

**Figure S4.** Inject of beetles laterally between the 4^th^ and 5^th^ abdominal segments using one.
